# Multidrug-Resistant *Corynebacterium striatum* Associated with Increased Use of Parenteral Antimicrobial Drugs

**DOI:** 10.3201/eid2211.160141

**Published:** 2016-11

**Authors:** William O. Hahn, Brian J. Werth, Susan M. Butler-Wu, Robert M. Rakita

**Affiliations:** Author affiliation: University of Washington, Seattle, Washington, USA

**Keywords:** Corynebacterium striatum, bacteria, antimicrobial resistance, multidrug resistance, parenteral antimicrobial drugs

## Abstract

*Corynebacterium striatum* is an emerging multidrug-resistant bacteria. We retrospectively identified 179 isolates in a clinical database. Clinical relevance, in vitro susceptibility, and length of parenteral antimicrobial drug use were obtained from patient records. For patients with hardware- or device-associated infections, those with *C. striatum* infections were matched with patients infected with coagulase-negative staphylococci for case–control analysis. A total of 87 (71%) of 121 isolates were resistant to all oral antimicrobial drugs tested, including penicillin, tetracycline, clindamycin, erythromycin, and ciprofloxacin. When isolated from hardware or devices, *C. striatum* was pathogenic in 38 (87%) of 44 cases. Patients with hardware-associated *C. striatum* infections received parenteral antimicrobial drugs longer than patients with hardware-associated coagulase-negative staphylococci infections (mean ± SD 69 ± 5 days vs. 25 ± 4 days; p<0.001). *C. striatum* commonly shows resistance to antimicrobial drugs with oral bioavailability and is associated with increased use of parenteral antimicrobial drugs.

## Abstract 2

Device-related infections with this pathogen frequently require prolonged parenteral therapy.

Corynebacteria are a normal component of the microbiota of human skin and mucous membranes. At the University of Washington Medical Center (Seattle, WA, USA), *Corynebacterium striatum* has historically been the second most commonly isolated *Corynebacterium* species, after *C. jeikium* (*1*). Although *C. striatum* is a frequent colonizer (*2*), it might also be implicated as a true pathogen when isolated in multiple samples from sterile sites or from indwelling hardware and devices (*2**–**9*). Determination of whether an isolate represents infection, colonization, or contamination is based upon clinical judgment.

Although early reports indicated that *C. striatum* isolates were frequently susceptible to many antimicrobial drugs, including β-lactams, tetracycline, and fluoroquinolones (*8**–**11*), more recent studies have demonstrated increasing multidrug resistance (*12**–**17*). To clarify the spectrum of disease associated with *C. striatum,* we retrospectively extracted clinical information for immunocompetent patients with *C. striatum* isolates to determine clinical relevance, antimicrobial drug susceptibilities, and length of parenteral therapy. For patients with device-associated infections, we compared the length of parenteral therapy for *C. striatum* with that for coagulase-negative staphylococci, other low-virulence organisms that commonly colonize the skin.

## Methods

### Patients

We used an algorithm-based query of the De-Identified Clinical Data Repository maintained by the Institute for Translational Health Sciences of the University of Washington (Seattle, WA, USA) to identify 213 patients from the university medical center infected with *C. striatum* isolated from a clinical sample during 2005–2014. Adult patients (>18 years of age) were included if *C. striatum* was isolated from a specimen submitted for bacterial culture and identified to the species level. Because we were interested in whether *C. striatum* would be considered pathogenic in an immunocompetent population, patients with active immunosuppression were excluded from our analysis. We excluded 34 patients with immunosuppressant use at the time of culture with microbiological growth on the basis of pharmacy records (defined as documentation of use of corticosteroids, methotrexate, infliximab, adalimumab, tacrolimus, cyclosporine, mycophenolate mofetil, or rituximab), or with a diagnosis of active malignancy or HIV/AIDS according to codes from the International Classification of Diseases, 9th Revision. Our algorithm-based method was confirmed by using a manual chart review.

### Bacterial Identification and In Vitro Drug Susceptibility Testing

Corynebacteria were identified to the species level if isolated in pure culture or if deemed to be clinically meaningful if present in a polymicrobial culture. Identification of corynebacteria was initially performed during 2005–2012 by using the RapID CB Plus Kit (Thermo Fisher Scientific, Waltham, MA, USA). This kit correctly identifies 95% of *Corynebacterium* isolates to the species level (*18*). During 2012–2014, corynebacteria were identified by using matrix- assisted laser desorption/ionization time-of-flight (MALDI-TOF) mass spectrometry (MALDI Biotyper and Biotyper software versions 3.0 and 3.1; Bruker Daltonics, Billerica, MA, USA) and a cutoff score of 2.0. Use of MALDI-TOF mass spectrometry with the Bruker system database correctly identifies corynebacteria to the genus level for >99% of isolates and correctly identified *C. striatum* in 100% (51/51) of clinical isolates tested (*19*).

Phenotypic susceptibility testing was performed by using the E-test (bioMérieux, Marcy l’Étoile, France) and Remel blood Mueller Hinton agar (Thermo Fisher Scientific) and incubation for 24–48 h at 35°C in ambient air, according to breakpoints of the Clinical and Laboratory Standards Institute (*20*). Because breakpoints have recently changed (*21*), we tested whether shifting the breakpoint would alter our results. Susceptibilities were available for penicillin, ciprofloxacin, clindamycin, erythromycin, and tetracycline. For patients from whom >1 isolate of *C. striatum* were obtained, we considered only the first isolate in our analysis.

### Clinical Data Extraction

We obtained the following variables from chart review by manual extraction: patient location when the culture was obtained (i.e., inpatient versus outpatient), whether the culture grew *C. striatum* in pure culture or was polymicrobial, whether the treating physician considered the isolate to be clinically relevant or a contaminant, length of parenteral therapy administered, and whether adverse events were documented. If the treating physician did not comment on the isolate, the isolate was categorized as clinically irrelevant. Adverse events were defined as clinical event necessitating a change in antimicrobial agent and were graded according to the Common Terminology for Adverse Events (*22*). Serious adverse events were considered grade 3 or 4. The outpatient parenteral antimicrobial therapy practices at the University of Washington Medical Center monitor for adverse events by weekly measurement of a complete blood count and monitoring of renal function. We used these weekly measurements as a proxy to verify ongoing administration of outpatient parenteral therapy in combination with review of documentation in clinical notes.

### Matched Case−Control Analysis

In a subset of patients with hardware-associated osteomyelitis or infections of implanted cardiac devices, we performed a matched case−control analysis to examine length of parenteral therapy in *C. striatum* cases compared with that for persons infected with coagulase-negative staphylococci (controls). Persons infected with coagulase-negative staphylococci were chosen as controls because isolates are frequently found in clinical samples (enabling appropriate matching), colonize the skin, and generally have low virulence, similar to *C. striatum*. Cases were matched to controls on the basis of age (± 5 years), site of infection, and presence or absence of hardware associated with the infection site.

### Statistical Analysis

Statistical analysis was performed by using Prism 6 (GraphPad Software Inc., La Jolla, CA, USA). Data are reported as mean ± SE. Parametric data were compared by using the *t*-test, and nonparametric data were compared by using χ^2^ or Mann-Whitney U tests. A p value <0.05 was considered significant.

## Results

We identified 179 immunocompetent adult patients infected with *C. striatum* isolated from clinical specimens. A substantial proportion (48%, 86/179) of these isolates were believed to be from clinically relevant infections. For comparison, in our laboratory system during a similar period, ≈42,000 isolates of *Staphylococcus aureus* and 4,800 isolates of coagulase-negative staphylococci were recovered with in vitro susceptibilities determined.

**Figure 1:**
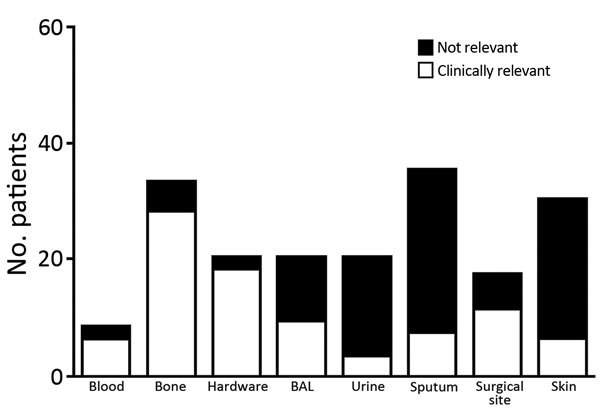
Patients infected with *Corynebacterium striatum*, by specimens isolated from a particular anatomic site, at the University of Washington Medical Center, Seattle, Washington, USA, 2005–2014. Hardware, surgical specimen obtained from a location anatomically in connection with a foreign device; BAL, bronchoalveolar lavage; urine, specimen isolated from a urine sample (we were unable to determine presence or absence of a catheter); surgical site, deep surgical specimen; skin, wound swab from a nonsurgical superficial specimen.

Consistent with previous reports that *C. striatum* is a colonizer of the skin and mucous membranes, isolates were frequently reported in sputum and skin samples (65/179) (Figure 1). Interpretation of clinical relevance varied markedly by sample site. Isolates from deep surgical specimens, such as bone and surgical hardware, were generally considered to be clinically relevant (88%–95%), whereas isolates from urine, sputum, or skin were rarely considered to be clinically relevant (10%–15%) (Figure 1). Isolates from bronchoalveolar lavage samples were indicated by clinicians to be clinically relevant in 45% (9/20) of cases, and all of these patients were empirically treated with vancomycin for healthcare-associated pneumonia for a mean ± SE duration of 11 ± 1.9 days. No clinical failures for vancomycin therapy were documented.

**Figure 2:**
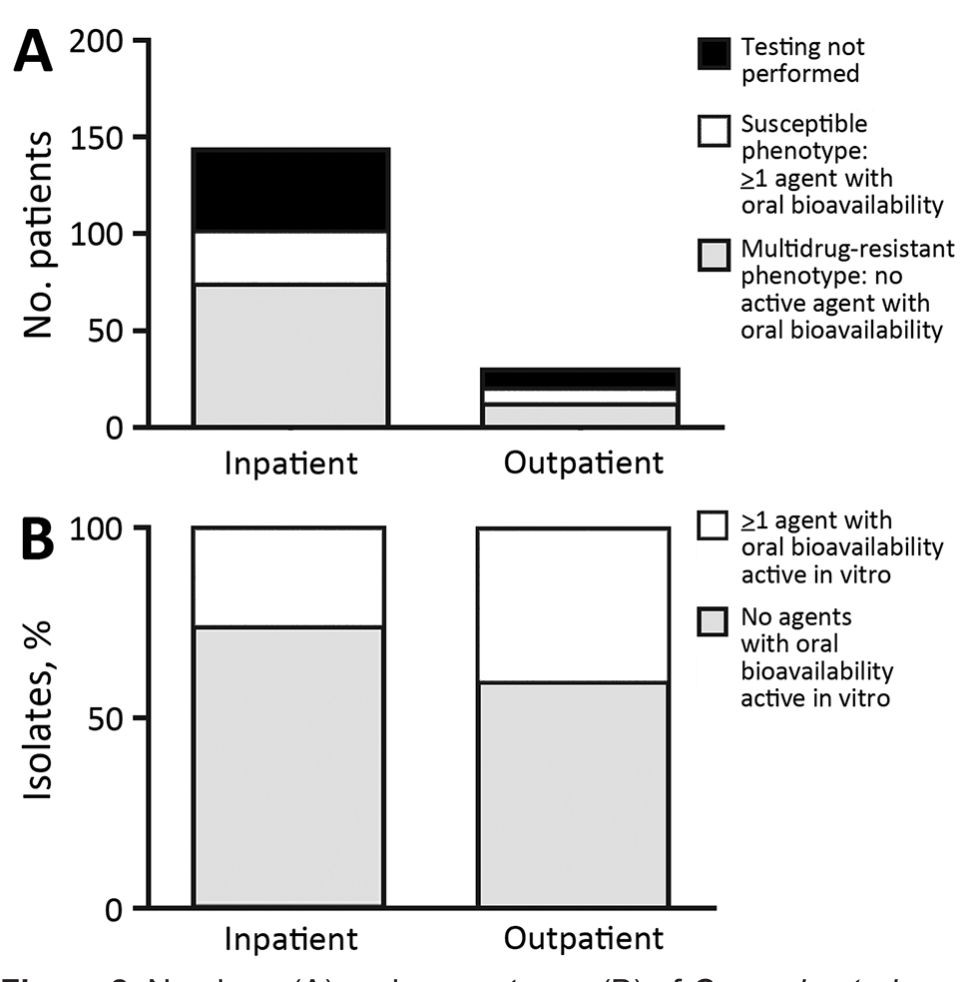
Numbers (A) and percentages (B) of *Corynebacterium striatum* isolates from patients at the University of Washington Medical Center, Seattle, Washington, USA, 2005–2014, with a multidrug-resistant phenotype for all antimicrobial drugs tested (penicillin, ciprofloxacin, clindamycin, erythromycin, and tetracycline). Inpatient or outpatient indicates clinical setting in which cultures were performed.

Eighty percent (143/179) of isolates were found in an inpatient setting. Susceptibility testing was performed for 121/179 isolates, and 72% (87/121) were resistant to all oral antimicrobial drugs tested (Figure 2). The percentage of drug-resistant isolates obtained from the inpatient setting did not differ from that found in the outpatient setting (p = 0.27).

We determined in vitro susceptibilities for several antimicrobial drugs (Table). In general, MIC distributions were bimodal, whereby most drugs had low MICs or high MICs, except for vancomycin, which was universally active in vitro. The breakpoint of the Clinical and Laboratory Standards Institute for penicillin changed in 2015 (*21*) from 1 mg/L to 0.125 mg/L. This change increased the rate of isolates considered penicillin resistant from 85% to 98%, but did not affect the number of isolates without an oral drug option because all isolates with a penicillin MIC <1.0 mg/L were susceptible to tetracycline. Daptomycin was rarely formally tested in our laboratory during this period (n = 6), including the 2 patients we previously described (*14*). Initial MICs for daptomycin were 0.032–0.125 mg/L, but it was rarely used by clinicians in our cohort. Similarly, linezolid was rarely tested (n = 10), was uniformly active in vitro, but was not used clinically.

**Table:** Drug resistance patterns for *Corynebacterium striatum* isolates from patients at the University of Washington Medical Center, Seattle, Washington, USA, 2005–2014*

Characteristic	Penicillin, n = 121	Tetracycline, n = 119	Clindamycin, n = 103	Erythromycin, n = 72	Ciprofloxacin, n = 119	Vancomycin, n = 120
Mean MIC	18	34	209	66	51	0.6
Median MIC	8	32	256	16	32	0.5
MIC range	0.125–256	0.125–256	0.25–256	0.25–256	0.125–256	0.125–1
MIC_50_	8	32	256	16	32	0.5
MIC_90_	32	64	256	256	32	1

*Resistance is defined as per Clinical and Laboratory Standards Institute Guidelines (*20*). Values are in milligrams/liter. MIC_50_, concentration required to inhibit 50% of bacteria; MIC_90_, concentration required to inhibit 90% of bacteria.

All patients with infections deemed to be clinically relevant were initially given vancomycin, except 1 patient with a documented allergy to vancomycin who received daptomycin. As expected, the length of time parenteral antimicrobial drugs were administered varied widely by anatomic site of infection and presence of a foreign body. Prosthetic joint infections were treated with parenteral therapy for 54 ± 7 days, and other hardware- or device-associated infections were treated for 65 ± 10 days. One patient with a ventricular assist device−associated infection who received vancomycin for 650 days was excluded from this analysis (outlier).

**Figure 3:**
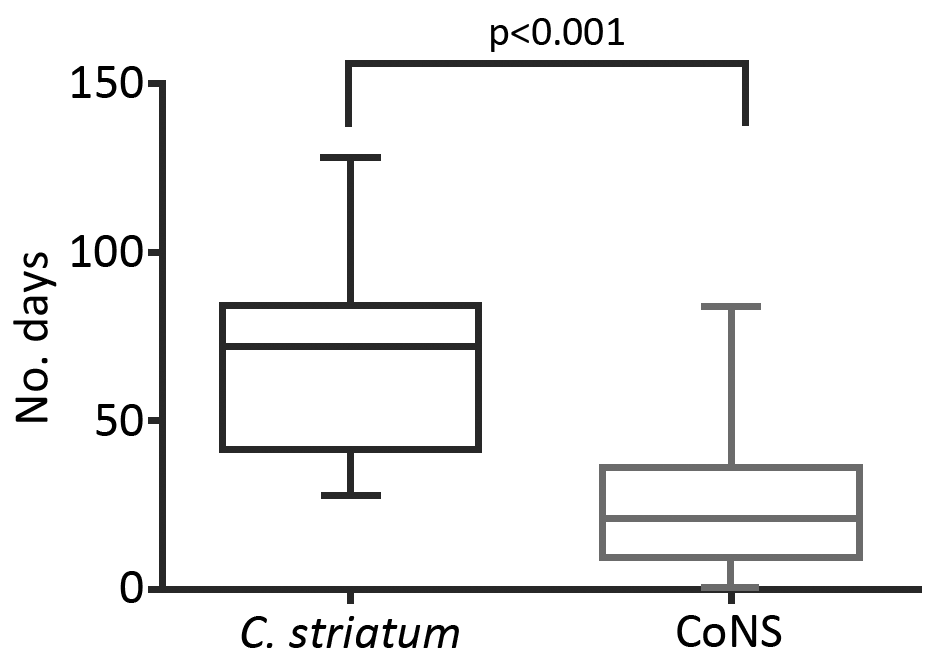
Length of use of parenteral intravenous antimicrobial drugs in matched case−control analysis of *Corynebacterium striatum* isolates and isolates of coagulase-negative staphylococci in patients with hardware-associated infections, University of Washington Medical Center, Seattle, Washington, USA, 2005–2014. Horizontal lines within boxes indicate median values, whiskers indicate minimum and maximum values, and boxes indicate 25th and 75th percentiles. Mean durations of parenteral antimicrobial drug use for patients infected with *C. striatum* and those infected with coagulase-negative staphylococci were compared by using the Mann-Whitney U test. CoNS, coagulase-negative staphylococci.

In a subset restricted to the 38 patients with hardware (including prosthetic joint) or device-associated infections deemed clinically meaningful, we successfully matched 27 patients with control patients infected with coagulase-negative staphylococci. Eleven patients could not be matched for site of infection or age. When we compared control patients infected with coagulase-negative staphylococci with patients infected with *C. striatum*, we found that those infected with *C. striatum* had a longer course of parenteral antimicrobial drugs (69 ± 5 days vs. 25 ± 4 days; p<0.001) (Figure 3). *C. striatum* isolates were more likely to be monomicrobial (14/27, 52%) than coagulase-negative staphylococci (5/27, 19%) (p = 0.02).

Five serious adverse events were associated with parenteral antimicrobial drugs in the *C. striatum* group and only 1 serious adverse event in the coagulase-negative staphylococci group (p = 0.19). The serious adverse events (all associated with vancomycin) were 1 drug reaction with eosinophilia and systemic symptoms syndrome, 2 acute kidney injuries with creatinine levels >3 times baseline values, and 3 absolute neutrophil counts <1,000 cells/mm^3^.

## Discussion

Because *C. striatum* can be a component of the skin microbiota, determination of whether microbiologic growth in a clinical sample represents an infection depends on clinical judgment. Previous investigations have described *C. striatum* primarily as a nosocomial pathogen, frequently in the setting of underlying malignancy or organ transplantation (*15**,**23**,**24*). Our report documents that clinicians encountering *C. striatum* in clinical samples of immunocompetent patients frequently consider the isolate to be a true pathogen.

Prior studies of small groups of patients have indicated that isolation of *C. striatum* from bone or a medical device is typically considered by the treating physician to be relevant (*24**–**26*), and our study confirms these findings. Furthermore, our study provides support for the pathogenic role of *C. striatum* in hardware-associated infections because we found that these infections with *C. striatum* were more likely to be monomicrobial than infections with coagulase-negative staphylococci. In contrast to some reports in which isolation from a respiratory sample was frequently determined to be clinically relevant (*15**,**27**,**28*), less than half of the respiratory isolates in our study were considered to reflect lower respiratory tract infections.

We documented that a multidrug-resistant phenotype of *C. striatum* directly affects clinical care. Osteomyelitis and hardware-associated infections are difficult to treat, often requiring a prolonged course of antimicrobial drugs. In some situations, guidelines recommend a limited duration of parenteral therapy followed by a longer period of oral therapy (*29*). A lack of well-tolerated oral treatment options active against *C. striatum* would be expected to lead to a longer duration of use of parenteral antimicrobial drugs for patients with these infections, and our matched case−control study confirmed this expectation.

The longer that parenteral antimicrobial therapy is necessary, the greater the likelihood of adverse events associated with intravenous access. These events include a rate of line events (mostly thrombosis) ranging from 5 to 17 episodes/100 devices and infection rates of 0.5 to 5 infections/100 lines (*30*). Although we documented more adverse events associated with treatment in the *C. striatum* group than in the coagulase-negative staphylococci group, this difference did not achieve a priori statistical significance, and we did not capture line thrombosis events. Furthermore, parenteral therapy is associated with substantially increased costs, even when comparing an inexpensive parenteral antimicrobial drug (vancomycin) with an expensive oral antimicrobial drug (linezolid) (*31*). In addition, parenteral options for *C. striatum* will be increasingly limited because our group and others have reported clinical failures caused by rapid development of high-level daptomycin resistance (*14**,**16**,**25*). Because daptomycin resistance can emerge rapidly, it is reasonable to assume that increased daptomycin use could also cause resistance to this drug.

One oral treatment option for multidrug-resistant *C. striatum* infections is linezolid. Although testing for *C. striatum* linezolid susceptibility is rarely performed in our clinical microbiology laboratory, to our knowledge, linezolid resistance has never been reported for corynebacteria. Nevertheless, linezolid is poorly tolerated during the long courses of treatment required for hardware- or device-associated infections and has shown a rate of adverse events leading to treatment discontinuation ranging from 34% to 80% (*32**,**33*). None of our patients in our study were treated with linezolid for a prolonged time, which most likely reflects reluctance of physicians to use an agent with such a high rate of toxicity.

We demonstrated that multidrug resistance was common even in isolates that were not considered to be clinically meaningful in an outpatient setting. We hypothesize that these resistant strains of *C. striatum* are probably circulating in the community rather than emerging under nosocomial pressure, but further studies would be needed to establish the ecologic niche of drug-resistant *C. striatum* strains.

Strengths of our study include a systems-wide approach to *C. striatum* infections in immunocompetent hosts and the large number of *C. striatum* case reports we reviewed. Detailed clinical information, including the treating physician’s interpretation of the sample, was linked to microbiological isolates. *C. striatum* will probably be recognized in more clinical settings because use of MALDI-TOF mass spectrometry enables *C. striatum* to be rapidly identified to the species level with a high degree of confidence without molecular techniques (*19*).

Limitations of our study include its retrospective nature and use of data from 1 health system, which restricted potential generalizability of the results. Given that our study was a retrospective study conducted over a 10-year period, we also cannot state whether the isolates are clonally related or reflect a diverse group with divergent mechanisms of drug resistance. In addition, we relied on the treating physician’s interpretation to determine the clinical relevance of an isolate. We have no other way of determining whether the isolate was causing disease, but we believe that this limitation is indicative of general clinical practice. Determining causality would require a different series of mechanistic investigations.

Our study demonstrates that *C. striatum* is an emerging multidrug-resistant pathogen. Our results highlight the need to identify corynebacteria to the species level, which is now readily performed by using MALDI-TOF mass spectrometry, and perform susceptibility testing for any isolate that is believed to be clinically meaningful. The frequent resistance of *C. striatum* to all easily tolerated oral antimicrobial drugs supports the need for development of new agents with good oral bioavailability and acceptable long-term safety profiles that are active against gram-positive organisms.
